# Exploring genes for immunoglobulin A nephropathy: a summary data-based mendelian randomization and FUMA analysis

**DOI:** 10.1186/s12920-023-01436-8

**Published:** 2023-01-29

**Authors:** Qian Zhang, Kang Zhang, Yining Zhu, Guangwei Yuan, Jingyun Yang, Minmin Zhang

**Affiliations:** 1 Department of Nephrology, Huashan Hospital, Fudan University, 12 Middle Wurumuqi Road, Jingan District, Shanghai, 200040 China; 2 Wuhu Hospital of Traditional Chinese Medicine, Anhui College of Traditional Chinese Medicine, Wuhu, Anhui China; 3 School of Mathematical Sciences, Fudan University, Yangpu District, Shanghai, China; 4 College of Professional Studies, Northeastern University, Boston, MA USA; 5 Rush Alzheimer’s Disease Center, Rush University Medical Center, 1750 W Harrison ST, STE 1000, Chicago, IL 60612 USA; 6 Department of Neurological Sciences, Rush University Medical Center, Chicago, IL USA

**Keywords:** Immunoglobulin A nephropathy, Expression quantitative trait loci, Summary data-based Mendelian randomization, Genome-wide association study, Functional mapping

## Abstract

**Background:**

Immunoglobulin A nephropathy (IgAN) is a complex autoimmune disease, and the exact pathogenesis remains to be elucidated. This study aimed to explore genes underlying the pathogenesis of IgAN.

**Methods:**

We conducted the summary data-based Mendelian randomization (SMR) analysis and performed functional mapping and annotation using FUMA to explore genetic loci that are potentially involved in the pathogenies of IgAN. Both analyses used summarized data of a recent genome-wide association study (GWAS) on IgANs, which included 477,784 Europeans (15,587 cases and 462,197 controls) and 175,359 East Asians (71 cases and 175,288 controls). We performed SMR analysis using Consortium for the Architecture of Gene Expression (CAGE) expression quantitative trait loci (eQTL) data and replicated the analysis using Genotype-Tissue Expression (GTEx) eQTL data.

**Results:**

Using the CAGE eQTL data, our SMR analysis identified 32 probes tagging 25 unique genes whose expression were pleiotropically associated with IgAN, with the top three probes being ILMN_2150787 (tagging *HLA-C*, *P*_SMR_= 2.10 × 10^–18^), ILMN_1682717 (tagging *IER3*, *P*_SMR_= 1.07 × 10^–16^) and ILMN_1661439 (tagging *FLOT1*, *P*_SMR_=1.16 × 10^–14^). Using GTEx eQTL data, our SMR analysis identified 24 probes tagging 24 unique genes whose expressions were pleiotropically associated with IgAN, with the top three probes being ENSG00000271581.1 (tagging *XXbac-BPG248L24.12*, *P*_SMR_= 1.44 × 10^–10^), ENSG00000186470.9 (tagging *BTN3A2*, *P*_SMR_= 2.28 × 10^–10^), and ENSG00000224389.4 (tagging *C4B*, *P*_SMR_= 1.23 × 10 ^–9^). FUMA analysis identified 3 independent, significant and lead SNPs, 2 genomic risk loci and 39 genes that are potentially involved in the pathogenesis of IgAN.

**Conclusion:**

We identified many genetic variants/loci that are potentially involved in the pathogenesis of IgAN. More studies are needed to elucidate the exact mechanisms of the identified genetic variants/loci in the etiology of IgAN.

**Supplementary Information:**

The online version contains supplementary material available at 10.1186/s12920-023-01436-8.

## Background

Immunoglobulin A nephropathy (IgAN), first described by Jean Berger in 1968 [1], is one of the most common forms of glomerulonephritis (GN) in the world [2]. It is characterized by the deposition of IgA immune complexes (specifically the IgA1 subclass) in the glomerular mesangium, leading to frequent episodes of hematuria and/or proteinuria [3]. Approximately 20–40% of IgAN patients will progress to end-stage renal disease (ESRD) within 10–20 years of diagnosis [4, 5], causing a critical public health burden.

IgAN is a complex autoimmune disease with contributions from multiple factors, such as preference of salty food [6] and a family history of chronic glomerulonephritis [7]. Previous studies also indicated the important role of genetics in the etiology of IgAN. The prevalence of IgAN varies considerably across ethnicities, being the highest in Asians, moderate in Caucasians and the lowest in the African population [8], implying that both environmental and genetic factors are likely to be involved in the pathogenesis of IgAN. Genome-wide association studies (GWAS) identified several independent risk alleles for IgAN in East Asians and Europeans, such as genetic loci in *CFH*, *TNFSF13, ST6GAL1* and *ACCS *[9–13]. Recent research also discovered two distinct genome-wide significant loci in *C1GALT1* and *C1GALT1C1* in association with defective O-glycosylation of serum immunoglobulin A1 (IgA1), the key pathogenic defect in IgAN [14]. However, the exact pathogenic mechanisms underlying the observed associations in general and the genetic associations in particular remains to be elucidated.

An important goal of public health is to identify modifiable risk factors of a disease or disorder to develop effective interventions and therapeutics. However, because of confounding, reverse causality, and selection bias, risk factors discovered by traditional observational epidemiology research were frequently found to be deceptive [15, 16]. Randomized controlled trials (RCTs) are often regarded as the gold standard for drawing causal conclusions because all the parameters, except for the exposure of interest, are comparable between the groups [17]. However, it is often time-consuming and expensive to conduct RCTs, and in some cases, allocation of exposure is either immoral or unfeasible.

Mendelian randomization (MR) refers to bioinformatical methods that assess pleiotropic effect of modifiable risk factors on a disease/disorder by utilizing proxy measures of these risk factors, thereby avoiding the necessity of conducting a conventional RCT [18]. In MR, instrumental variables (IVs) are used as a proxy for randomizing individuals to ensure comparable results regardless of known/unknown confounding factors. Due to the random allocation of alleles during gamete formation, which occurs well before the exposure or outcome, genetic variants are often used as the IVs. Pleiotropic associations can be estimated from MR because inherited genetic variants are independent of potential confounding factors. By using MR, confounding and reverse causation, which are commonly encountered in traditional association studies, can be greatly minimized. MR has been successful in identifying gene expression or DNA methylation sites showing pleiotropic association with various phenotypes, such as systemic lupus erythematosus (SLE), educational attainment, and severity of COVID-19 [19–21]. In this study, we adopted the summary data-based MR (SMR) approach integrating summarized cis-expression quantitative trait loci (cis- eQTL) data and GWAS data for IgAN to prioritize genes whose expressions are pleiotropically associated with IgAN, with gene expression being the exposure and IgAN being the outcome.

Although previous genetic studies have identified independent risk alleles for IgAN, more studies are needed to better understand the genetic mechanisms underlying IgAN, such as the roles of the non-coding regulatory regions. FUMA is an Internet-based program that utilizes multiple biological databases to provide an easy-to-use tool for functional mapping and annotation of genetic variants identified in GWAS [22]. FUMA can provide multiple post-GWAS analysis simultaneously, including functional annotation of candidate SNPs, gene mapping, tissue-expression analysis of the prioritized genes, gene set enrichment analysis (GSEA), and interactive visualization. Previously research indicated that FUMA validated known candidate genes and identified additional putative genes through eQTL mapping and chromatin interaction mapping [22], providing valuable clues for understanding the complex genetic mechanisms underlying a disease/disorder. Therefore, we also conducted FUMA analysis to further explore genetic variants and genomic loci in the pathogenesis of IgAN.

## Methods

### GWAS data for IgAN

The GWAS summarized data for IgAN were provided by a recent genome-wide association meta-analysis of IgAN [23]. The results were based on meta-analyses of IgAN using data from three population-based projects: The BioBank Japan (BBJ) [24], the UK Biobank [25], and GWAS summary results from FinnGen (https://www.finngen.fi/), with the sample size being 175,359 (71 cases and 175,288 controls), 344,365 (15,418 cases and 328,947 controls), and 133,419 (169 cases and 133,250 controls) for the three projects, respectively. As a result, the meta-analysis included 477,784 Europeans (15,587 cases and 462,197 controls) and 175,359 East Asians (71 cases and 175,288 controls). IgAN was diagnosed based on International Classification of Diseases, 10th revision (ICD-10). The three projects used different genotyping flatforms and reference panels for imputation. GWAS analysis was done using generalized mixed-effects models, with adjustment of different covariates and principal components (PCs) (Additional file 1: Table S1). The GWAS summarized data can be downloaded at http://ftp.ebi.ac.uk/pub/databases/gwas/summary_statistics/GCST90018001-GCST90019000/GCST90018866/harmonised/.

## eQTL data for SMR analysis

In the SMR analysis, cis-eQTL genetic variants were used as the IVs for gene expression. cis-eQTLs were defined as the eQTLs that are not more than 5 Mb away from the probes. A default cis window of 2000 kb was used. We performed separate SMR analysis using eQTL data from two sources. Specifically, we used the CAGE eQTL summarized data for whole blood, which included 2765 participants of European ancestry [26]. To see whether the significant findings can be replicated, we also performed separate SMR analysis using the V7 release of the GTEx eQTL summarized data for whole blood, which included 338 participants of European ancestry [27]. The eQTL data can be downloaded at https://cnsgenomics.com/data/SMR/#eQTLsummarydata. We did not use GTEx eQTL data from kidney due to the extremely limited sample size (e.g., n = 73 for kidney cortex and n = 4 for kidney medulla).

## SMR analysis

**Fig. 1:**
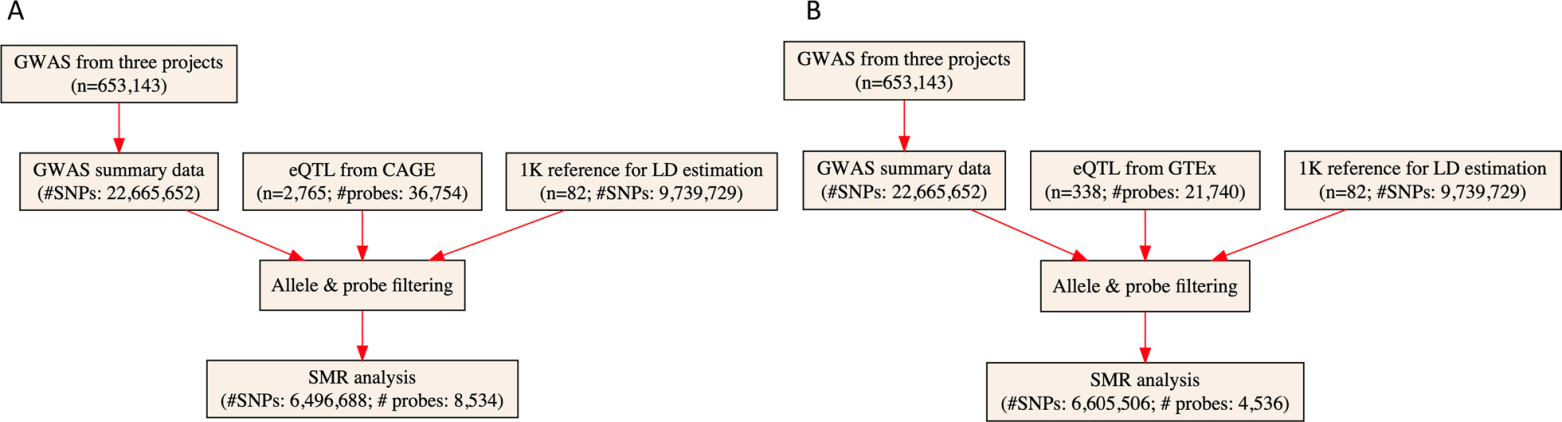
Flow chart for the SMR analysis. **A** SMR analysis using eQTL data from CAGE; and **B** SMR analysis using eQTL data from GTEx. eQTL, expression quantitative trait loci; GWAS, genome-wide association studies; LD, linkage disequilibrium; SMR, summary data-based Mendelian randomization; SNP, single nucleotide polymorphisms

We conducted the SMR analysis as implemented in the software SMR, with cis-eQTL as the IV, gene expression as the exposure, and IgAN as the outcome. Detailed information regarding the SMR method can be found in a previous publication [28]. We conducted the heterogeneity in dependent instruments (HEIDI) test to evaluate the existence of linkage in the observed association. HEIDI uses multiple SNPs in a cis-eQTL region to distinguish pleiotropy from linkage. The null hypothesis is that there is a single causal variant underlying the observed association between gene expression and a trait. Testing against the null hypothesis is equivalent to testing whether there is heterogeneity in the estimated SMR effect for the SNPs in the cis-eQTL region. We adopted the default *P*_HEIDI_< 0.05 to indicate the existence of pleiotropy (i.e., the observed association could be due to two distinct genetic variants in high linkage disequilibrium with each other), which is a conservative approach as it retains fewer genes than when correcting for multiple testing. We adopted the default settings in SMR (Additional file 1: Table S2) and used false discovery rate (FDR) to adjust for multiple testing. The SMR analytic process is illustrated in Fig. 1.

## FUMA analysis

We conducted a FUMA analysis to functionally map and annotate the genetic associations to better understand the genetic mechanisms underlying IgAN. FUMA uses GWAS association results as the input and integrates information from multiple resources. It provides a friendly on-line platform for easy implementation of post-GWAS analysis, such as functional annotation and gene prioritization [22]. FUMA provides two major functions: SNP2GENE for annotating SNPs regarding their biological functions and SNP-to-genes mapping; and GENE2FUNC for annotating the mapped genes in biological contexts. In SNP2GENE, we performed both positional mapping (maximum distance 10 kb) and eQTL mapping (cis-eQTL, i.e., up to 1 Mb) using GTEx v8 of whole blood and kidney. We adopted the default settings otherwise for both SNP2GENE (e.g., maximum *P* value of lead SNPs being 5 × 10^− 8^ and r^2^ threshold for independent significant SNPs being 0.6) and GENE2FUNC (e.g., using FDR to correct for multiple testing in the gene-set enrichment analysis).

It is noteworthy that although both FUMA and SMR utilized eQTL information, the two methods attempted to explore the pathogenesis of IgAN through different perspectives: The SMR analysis tried to reveal gene expressions showing pleiotropic association with IgAN while FUMA attempted to pinpoint most likely relevant genetic variants in association with IgAN while taking into account the regional linkage disequilibrium (LD) patterns based on positional and eQTL information of the SNPs. Moreover, FUMA not only provided enrichment analysis of the prioritized genes in biological pathways and functional categories, it also revealed potential risk loci along the chromosomes.

Data cleaning and statistical/bioinformatical analysis was performed using R version 4.1.2 (https://www.r-project.org/), PLINK 1.9 (https://www.cog-genomics.org/plink/1.9/), SMR (https://cnsgenomics.com/software/smr/), and FUMA (https://fuma.ctglab.nl/).

## Results

### Basic information of the summarized data

The GWAS summarized data included a total of 22,665,652 SNPs. A total of 6437 SNPs were significantly associated with IgAN using the conventional P = 5 × 10^− 8^ as the cut-off. The GWAS meta-analysis of IgAN identified eight significant genetic loci (Additional file 1: Table S3). After checking allele frequencies among the datasets and linkage disequilibrium (LD) pruning, we found that there were more than 6 million eligible SNPs in each SMR analysis. The CAGE eQTL has a much larger number of participants than that of the GTEx eQTL data (2765 vs. 70), so is the number of eligible probes (8534 vs. 4536). In the FUMA analysis, about 8.6 million SNPs were used as input. The detailed information is shown in Table 1.

**Table 1:** Basic information of the eQTL and GWAS data

Data source	Effective number of participants*	Number of eligible genetic variants or probes
eQTL data		
CAGE	2765	8534
GTEx	338	4536
GWAS data for SMR analysis (case/control)		
BBJ	71/175,288	–
UKBB	15,418/328,947	–
FinnGen	169/133,250	–
Total	15,658/637,485	CAGE: 6,496,688; GTEx: 6,605,506
GWAS data for FUMA analysis (case/control)	15,658/637,485	22,665,652

*GWAS* genome-wide association studies, *QTL* quantitative trait loci, *BBJ* BioBank Japan, *UKBB* UK Biobank

*Number of participants for the corresponding eQTL data or the corresponding projects

## Pleiotropic association with IgAN

**Fig. 2:**
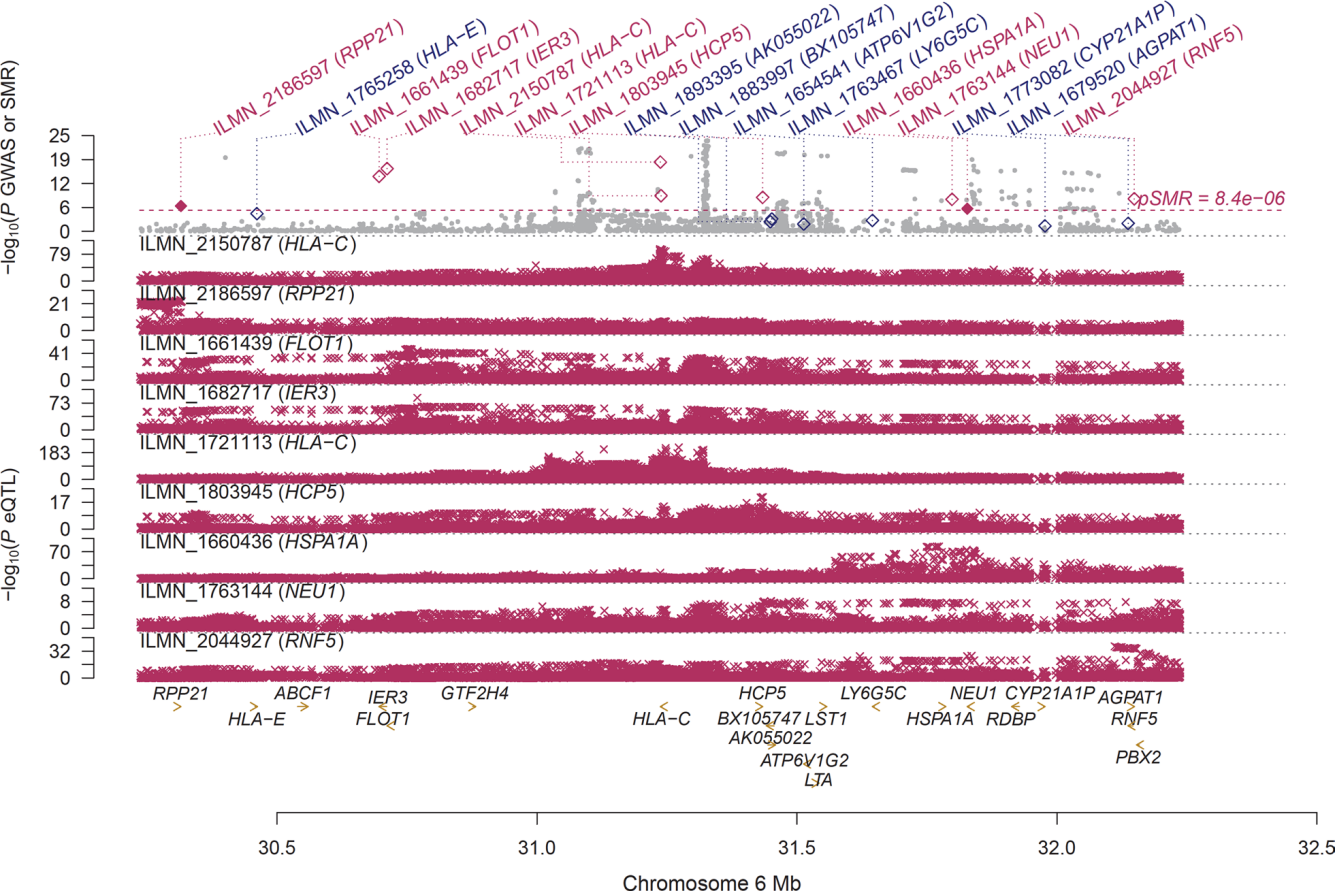
Pleiotropic association of *HLA-C* with IgAN. Top plot, grey dots represent the −log10(*P* values) for SNPs from the GWAS of IOP, with solid rhombuses indicating that the probes pass HEIDI test. Middle plot, eQTL results. Bottom plot, location of genes tagged by the probes; GWAS, genome-wide association studies; SMR, summary data-based Mendelian randomization; HEIDI, heterogeneity in dependent instruments; eQTL, expression quan-titative trait loci; IgAN, immunoglobulin A nephropathy

**Fig. 3:**
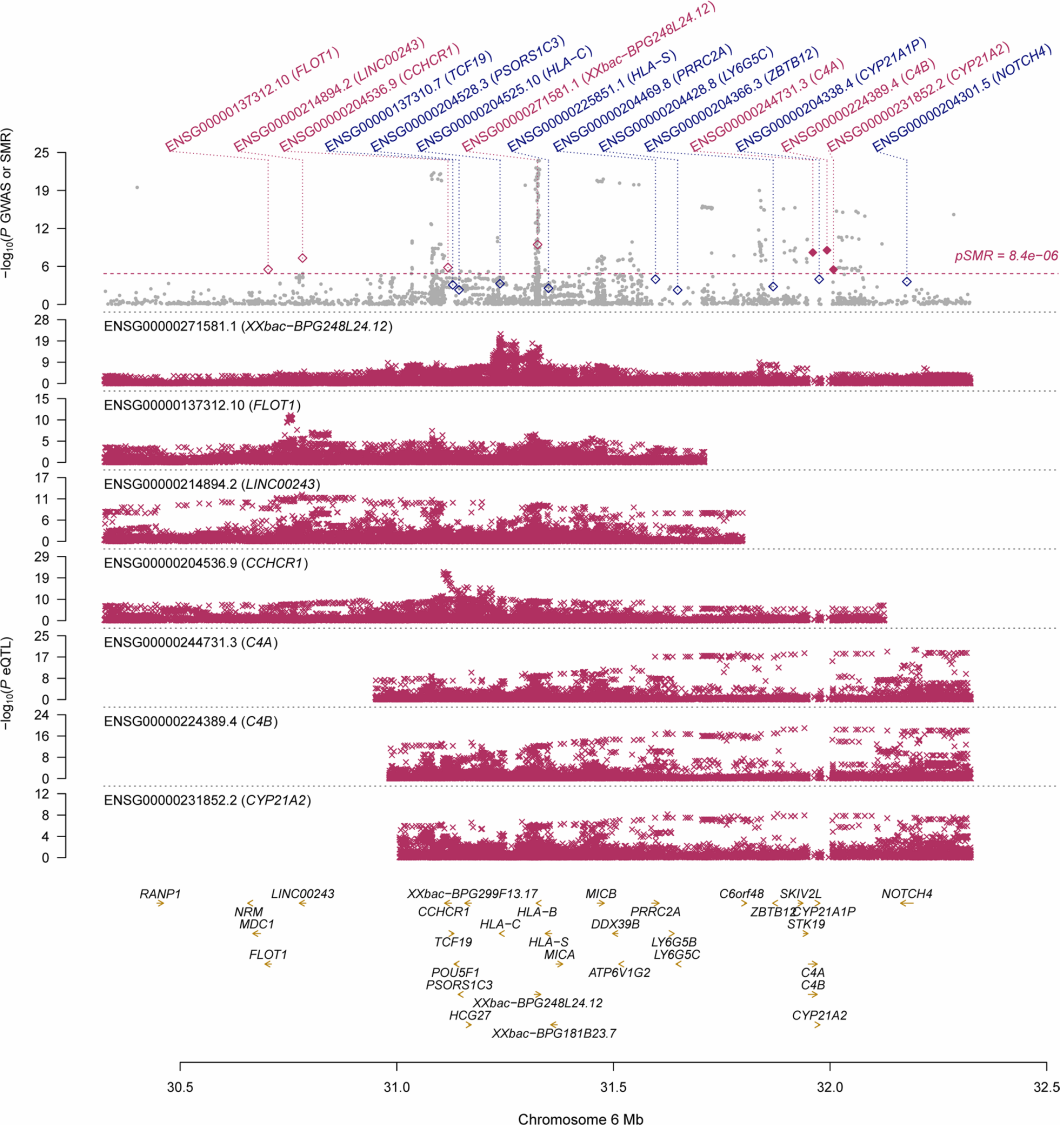
Pleiotropic association of *XXbac-BPG248L24.12*, *C4A* and *C4B* with IgAN. Top plot, grey dots represent the −log10(*P* values) for SNPs from the GWAS of IOP, with solid rhombuses indicating that the probes pass HEIDI test. Middle plot, eQTL results. Bottom plot, location of genes tagged by the probes. GWAS, genome-wide association studies; SMR, summary data-based Mendelian randomization; HEIDI, heterogeneity in dependent instruments; eQTL, expression quan-titative trait loci; IgAN, immunoglobulin A nephropathy

Using the CAGE eQTL data, our SMR analysis identified 32 probes tagging 25 unique genes whose expressions were pleiotropically associated with IgAN, with the top three probes being ILMN_2150787 (tagging *HLA-C*, *P*_SMR_= 2.10 × 10^–18^), ILMN_1682717 (tagging *IER3*, *P*_SMR_= 1.07 × 10^–16^) and ILMN_1661439 (tagging *FLOT1*, *P*_SMR_=1.16 × 10^–14^; Fig. 2; Table 2, Additional file 1: Table S4). Using GTEx eQTL data, our SMR analysis identified 24 probes tagging 24 unique genes whose expressions were pleiotropically associated with IgAN, with the top three probes being ENSG00000271581.1 (tagging *Xxbac-BPG248L24.12*, *P*_SMR_=1.44 × 10^–10^), ENSG00000186470.9 (tagging *BTN3A2*, *P*_SMR_=2.28 × 10^–10^), and ENSG00000224389.4 (tagging *C4B*, *P*_SMR _= 1.23 × 10^− 9^; Fig. 3; Table 2, Additional file 1: Table S4). Note that of the 25 unique genes identified in the SMR analysis using CAGE eQTL data, four genes (*FLOT1*, *BTN3A2*, *HLA-DRB6*, and *HLA-DRB1*) were also replicated in the SMR analysis using GTEx eQTL data (Additional file 1: Table S4).

**Table 2:** The top hit probes identified in SMR analyses

eQTL data	Probe	Gene	CHR	Top SNP	OR (95% CI)	P_eQTL_	P_GWAS_	Beta	SE	P_SMR_	P_HEIDI_	Q value
CAGE	ILMN_2150787	*HLA-C*	6	rs2523578	1.14 (1.11–1.17)	1.55 × 10^− 65^	2.62 × 10^− 24^	− 0.2378	0.0272	2.10 × 10^− 18^	9.48 × 10^− 12^	5.73 × 10^− 13^
	ILMN_1682717	*IER3*	6	rs2233980	0.85 (0.83–0.88)	2.05 × 10^− 60^	5.47 × 10^− 22^	− 0.2065	0.0249	1.07 × 10^− 16^	1.89 × 10^− 03^	7.30 × 10^− 12^
	ILMN_1661439	*FLOT1*	6	rs3130985	0.85 (0.83–0.88)	6.93 × 10^− 37^	2.55 × 10^− 22^	− 0.2666	0.0345	1.16 × 10^− 14^	2.68 × 10^− 02^	3.53 × 10^− 10^
	ILMN_1700067	*BTN3A2*	6	rs9393710	0.89 (0.86–0.92)	9.06 × 10^− 237^	3.86 × 10^− 12^	0.0832	0.0123	1.24 × 10^− 11^	9.13 × 10^− 08^	2.11 × 10^− 07^
	ILMN_1820787	*BTN2A1*	6	rs3734544	0.91 (0.88–0.94)	3.34 × 10^− 189^	1.40 × 10^− 10^	− 0.0877	0.0140	3.60 × 10^− 10^	1.40 × 10^− 08^	3.93 × 10^− 06^
	ILMN_1721113	*HLA-C*	6	rs9266075	1.08 (1.05–1.1)	2.99 × 10^− 183^	5.90 × 10^− 10^	− 0.0867	0.0143	1.35 × 10^− 09^	1.12 × 10^− 03^	1.02 × 10^− 05^
	ILMN_1803945	*HCP5*	6	rs2596495	0.85 (0.83–0.88)	5.73 × 10^− 14^	1.58 × 10^− 21^	0.4716	0.0798	3.48 × 10^− 09^	3.20 × 10^− 02^	1.94 × 10^− 05^
	ILMN_2044927	*RNF5*	6	rs192471087	0.91 (0.89–0.94)	2.49 × 10^− 37^	1.28 × 10^− 10^	− 0.1974	0.0343	8.59 × 10^− 09^	1.29 × 10^− 04^	3.00 × 10^− 05^
	ILMN_1804571	*ZKSCAN4*	6	rs13200462	0.89 (0.86–0.92)	2.61 × 10^− 19^	1.37 × 10^− 13^	− 0.3193	0.0558	1.08 × 10^− 08^	1.88 × 10^− 02^	3.00 × 10^− 05^
	ILMN_1660436	*HSPA1A*	6	rs494620	1.07 (1.05–1.1)	1.40 × 10^− 74^	1.81 × 10^− 09^	0.1417	0.0248	1.10 × 10^− 08^	2.16 × 10^− 09^	3.00 × 10^− 05^
GTEx	ENSG00000271581.1	*XXbac-BPG248L24.12*	6	rs9266244	1.14 (1.11–1.17)	1.52 × 10^− 16^	2.96 × 10^− 24^	0.2043	0.0319	1.44 × 10^− 10^	4.34 × 10^− 07^	6.22 × 10^− 06^
	ENSG00000186470.9	*BTN3A2*	6	rs68112369	0.89 (0.86–0.92)	9.15 × 10^− 70^	1.14 × 10^− 11^	0.1088	0.0172	2.28 × 10^− 10^	1.07 × 10^− 03^	6.22 × 10^− 06^
	ENSG00000224389.4	*C4B*	6	rs1270942	0.87 (0.84–0.9)	3.63 × 10^− 19^	1.44 × 10^− 16^	− 0.1233	0.0203	1.23 × 10^− 09^	5.84 × 10^− 02^	1.48 × 10^− 05^
	ENSG00000244731.3	*C4A*	6	rs116667074	0.87 (0.84–0.9)	5.15 × 10^− 19^	1.67 × 10^− 15^	0.1382	0.0233	2.88 × 10^− 09^	1.08 × 10^− 01^	1.96 × 10^− 05^
	ENSG00000214894.2	*LINC00243*	6	rs3094222	0.85 (0.83–0.88)	8.03 × 10^− 12^	3.93 × 10^− 22^	− 0.3086	0.0553	2.38 × 10^− 08^	5.63 × 10^− 03^	1.04 × 10^− 04^
	ENSG00000204644.5	*ZFP57*	6	rs2747431	0.93 (0.91–0.96)	2.03 × 10^− 85^	5.51 × 10^− 08^	− 0.0566	0.0108	1.62 × 10^− 07^	7.31 × 10^− 05^	4.88 × 10^− 04^
	ENSG00000204536.9	*CCHCR1*	6	rs1265087	0.93 (0.91–0.95)	4.64 × 10^− 22^	1.01 × 10^− 08^	− 0.2081	0.0423	8.60 × 10^− 07^	7.49 × 10^− 04^	1.91 × 10^− 03^
	ENSG00000137312.10	*FLOT1*	6	rs3094222	0.85 (0.83–0.88)	3.74 × 10^− 08^	3.93 × 10^− 22^	− 1.3396	0.2802	1.74 × 10^− 06^	1.11 × 10^− 02^	2.49 × 10^− 03^
	ENSG00000231852.2	*CYP21A2*	6	rs7763805	0.89 (0.86–0.92)	2.24 × 10^− 10^	5.15 × 10^− 13^	− 0.1665	0.0349	1.85 × 10^− 06^	3.40 × 10^− 01^	2.49 × 10^− 03^
	ENSG00000204622.6	*HLA-J*	6	rs1611336	1.07 (1.04–1.09)	6.54 × 10^− 20^	8.13 × 10^− 08^	0.1162	0.0251	3.78 × 10^− 06^	4.10 × 10^− 04^	4.10 × 10^− 03^

For a full list of the significant probes identified in the SMR analyses, please see Additional file 1: Table S4. The GWAS summarized data were provided by the study of Sakaue et al. and can be downloaded at http://ftp.ebi.ac.uk/pub/databases/gwas/summary_statistics/GCST90018001-GCST90019000/GCST90018866/harmonised/. The CAGE and GTEx eQTL data can be downloaded at https://cnsgenomics.com/data/SMR/#eQTLsummarydata.

OR (95% CI) is the odds ratio and the corresponding 95% confidence interval for the top SNPs. *P*_eQTL_ is the *P* value of the top associated cis-eQTL in the eQTL analysis, and P_GWAS_ is the *P* value for the top associated cis-eQTL in the GWAS analysis. Beta is the estimated effect size in SMR analysis, SE is the corresponding standard error, *P*_SMR_ is the *P* value for SMR analysis and P_HEIDI_ is the *P* value for the HEIDI test

*CAGE* Consortium for the Architecture of Gene Expression, *CHR* chromosome, *CI* confidence interval, *eQTL* expression quantitative trait loci, *GTEx* Genotype-Tissue Expression, *HEIDI* heterogeneity in dependent instruments, *OR* odds ratio, *SNP* single-nucleotide polymorphism, *SMR* summary data-based Mendelian randomization, *FDR* false discovery rate, *GWAS* genome-wide association studies, *IgAN* immunoglobulin A nephropathy

## Functional mapping and annotation

**Fig. 4:**
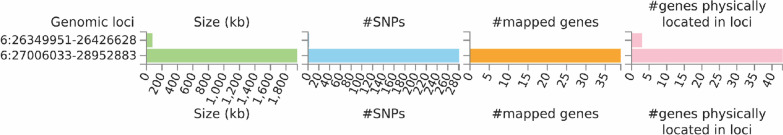
Genetic risk loci identified by FUMA analysis using GWAS data on IgAN. Genomic risk loci are displayed in the format of ‘chromosome:start position-end position’ on the Y axis. For each genomic locus, histograms from left to right depict the size, the number of candidate SNPs, the number of mapped genes (using positional mapping and eQTL mapping), and the number of genes known to be located within the genomic locus, respectively. eQTL, expression quantitative trait loci; GWAS, genome-wide association studies; SNP, single nucleotide polymorphism; IgAN, immunoglobulin A nephropathy

FUMA analysis identified 3 independent, significant and lead SNPs (rs2076030, rs469228, and rs1884937; Additional file 1: Tables S5–S7), and 2 genomic risk loci (Fig. 4; Additional file 1: Table S8). All the three SNPs are located on chromosome 6. In addition, FUMA identified 39 genes that are potentially involved in the pathogenesis of IgAN (Additional file 1: Table S9), which are clustered in one genomic risk locus, with the other genomic locus containing no identified genes (Fig. 4 & Additional file 1: Table S9). Of the 39 identified genes, four were also identified by SMR analysis using CAGE eQTL data, including *HIST1H2BK*, *ZSCAN16*, *ZKSCAN4*, and *ZKSCAN3*; and one (*TRIM27*) was identified by SMR analysis using GTEx eQTL data. Expression of the prioritized genes in 54 tissues can be found in Additional file 1: Table S10 and Figure S1.

GSEA was undertaken to test the possible biological mechanisms of the 39 candidate genes implicated in IgAN (Additional file 1: Table S11). A total of 310 gene sets with an adjusted *P* < 0.05 were identified. We found enrichment signals in intestinal immune network such as SLE (adjusted P = 9.00 × 10^–23^) as revealed by a recent GWAS study [13], and chromatin-related pathways such as chromatin assembly (adjusted P = 5.58 × 10^–11^) which are crucial regulators in cellular immunity[29].

## Discussion

In this study, we conducted SMR and FUMA analysis to prioritize genetic loci associated with IgAN. We identified multiple genetic variants, genes, gene sets and two genomic loci that may be involved in the pathogenesis of IgAN. These findings provided helpful leads to a better understanding of the etiology of IgAN.

We found that multiple genes in the human leukocyte antigen (HLA) complex whose expressions showed significantly pleiotropic association with IgAN using CAGE and/or GTEx eQTL data, such as *HLA-A/C/E/H/J/L*, *HLA-DQA1/A2*, and *HLA-DRB1/B6*. The HLA complex, known as major histocompatibility complex (MHC), plays important roles in enabling the immune system to recognize “self” versus “non-self” antigens. The first association between HLA and renal disease was reported more than 50 years ago. Since then, mounting evidence has demonstrated the importance of the HLA complex in IgAN [30]. Many genetic variants in the HLA complex have been found to be associated with the risk of IgAN [31–33]. Previous GWAS studies also identified a few genetic variants in MHC in association with IgAN in individuals of European and East-Asian ancestry [9, 11–13]. A recent study found that the expression of *HLA-DQB1* and *HLA-DRB1* decreased on the peripheral blood lymphocytes (PBLs) in IgAN patients, compared with the controls, and that abnormal HLA-DQB1 and HLA-DRB1 expression may aggravate the progression of IgAN [34], suggesting the possible involvement of the abnormal expression of both genes in the pathogenesis of IgAN. Abnormal mRNA expression of some HLA genes has been observed in many autoimmune diseases such as lupus and was found to be related to DNA methylation [35, 36]. It should be noted that HEIDI test was significant for some of the HLA genes except *HLA-DRB6*, *HLA-DQA1*, *HLA-DRB1*, *HLA-DQA2* (Additional file 1: Tabel S4), indicating the existence of pleiotropy. These findings indicated that inflammation and DNA methylation might be two possible mechanisms underlying the HLA’s involvement in IgAN. More studies are needed to elucidate the exact functions of HLA genes in the pathogenesis of IgAN.

In SMR analysis, we also found that two genes in the complement component C4 family, including *C4A* and *C4B*, whose expressions showed significantly pleiotropic association with IgAN using GTEx eQTL data (Table 2). Both genes are mapped in III region of the MHC on chromosome 6p21.3 [37]. The two genes, together with three other neighboring genes including *RP* (serine–threonine kinase), *CYP21* (steroid 21-hydroxylase) and *TNX* (tenascin-X), form a genetic unit called RCCX module (RP-C4A-CYP21-TNX or RP-C4B-CYP21-TNX) which determines gene copy number (GCN) variation [38]. GCN of the two genes was found to be associated with many autoimmune diseases. For example, a previous meta-analysis found that low *C4A* GCNs were associated with increased risk of SLE in Caucasian populations [39]. The expression of *C4A* and *C4B* was significantly upregulated in glomeruli of patients with IgAN [40]. The whole complement system can be activated by three pathways, including the classical pathway, the lectin binding pathway, and the alternative pathway, and *C4A* and *C4B* are likely involved in the classical pathway [41]. However, the exact roles of the two genes and the mechanisms underlying the pathogenesis of IgAN remain to be explored.

Our SMR analyses were based on three core assumptions: (1) The genetic variants used as IVs were associated with gene expression (i.e., concern of weak IV); (2) The genetic variants were not associated with confounders that bias the association of gene expression with IgAN; and (3) The genetic variants are related with IgAN only through their association with gene expression. Concerns about these assumptions are minimal, moderate, or cannot be verified directly. For Assumption 1, the SMR analysis assumed a *P* value threshold of 5 × 10^− 8^ to select the top associated eQTL. Therefore, the selected genetic variants indeed showed strong association with gene expression, and we believe that the concern about weak IV is minimal. The basis for Assumption 2 is that the genetic variants are not associated with socioeconomic and behavioural traits that commonly confound the effect of exposure (i.e., gene expression) on outcome (i.e., IgAN risk). Our SMR analyses used summarized data; therefore, we did not have data to directly test this assumption. Assumption 3 is regarding horizontal pleiotropy which can skew the MR results. Recent research indicated that horizontal pleiotropy was detectable in more than 48% of significant MR causalities, yielding an average bias of −131% to 201% in MR estimates. The existence of horizontal pleiotropy can induce false-positive causal findings in up to 10% of relationships [42]. For some of the identified genes, we did notice that the HEIDI test was significant, indicating that we should interpret these results with caution.

Our study has some limitations. The number of eligible probes and the sample size of the eQTL for the SMR analysis was limited, especially the GTEx eQTL. Moreover, we used FDR to correct for multiple testing. Together, we may miss some important genes that were not tagged in the eQTL data or filtered out by FDR. The HEIDI test indicated the existence of horizontal pleiotropy for some of the observed associations (Additional file 1: Table S4). Our SMR analysis used eQTL data from the blood as the sample size for eQTL data in kidney is rather limited in GTEx V7. It would be interesting to explore whether the findings still hold using kidney eQTL data which are based on larger sample sizes. Moreover, the GWAS data are based on mixed ancestries including Europeans and East Asians. We did not perform SMR analysis in subjects of European ancestry because the GWAS summarized data for European ancestry are not publicly available. In our SMR analyses, the exposure is gene expression which may be influenced by genetic variants. The SMR analyses, however, cannot distinguish between pleiotropy and causality. We did not perform genetic colocalization analysis [43], which aims to assess whether two traits are affected by the same or distinct causal variants and therefore serves as a good complement to the SMR analysis. We used the default setting in the SMR analyses; therefore, we only examined pleiotropic association in cis regions but not in trans regions. Future studies are needed to explore genes in trans regions in pleiotropic association with IgAN. The SMR approach adopted in this paper used a single instrument. Traditional methods, such as SMR, MR-Egger and median based regression, only make use of a single SNP instrument or multiple independent SNP instruments. Some new MR-based methods have been recently developed, such as the probabilistic Mendelian randomization method named PMR-Egger which can accommodate multiple correlated instruments and can control horizontal pleiotropic effects [44]. PMR-Egger was found to yield calibrated *P *values across a wide range of scenarios and improve the power of MR analysis over existing approaches, potentially leading to better replication and experimental validation on the top identified genes. Future studies that use these novel methods are needed to validate our findings.

## Conclusion

In summary, we performed SMR and FUMA analysis and identified many genetic variants/loci that are potentially involved in the pathogenesis of IgAN. More studies are needed to elucidate the exact mechanism of the identified genetic variants/loci in the etiology of IgAN.

## Supplementary Information

**Additional file 1.** Basic information of the GWAS summary data, the default parameters of SMR analysis, and supplementary results.

## Data Availability

The datasets supporting the conclusions of this article are available at https://cnsgenomics.com/data/SMR/#eQTLsummarydata, and http://ftp.ebi.ac.uk/pub/databases/gwas/summary_statistics/GCST90018001-GCST90019000/GCST90018866/harmonised/.

## Abbreviations

BBJ: BioBank Japan
CAGE: Consortium for the architecture of gene expression
eQTL: Expression quantitative trait loci
ESRD: End-stage renal disease
FDR: False discovery rate
GN: Glomerulonephritis
GWAS: Genome-wide association study
GSEA: Gene-set enrichment analysis
GTEx: Genotype-tissue expression
HEIDI: Heterogeneity in dependent instruments
HLA: Human leukocyte antigen
IgAN: Immunoglobulin A nephropathy
LD: Linkage disequilibrium
MHC: Major histocompatibility complex
MR: Mendelian randomization
RCT: Randomized controlled trial
SLE: Systemic lupus erythematosus
SMR: Summary data-based Mendelian randomization
